# Ictal and interictal SPECT with ^99m^Tc‐HMPAO in presurgical epilepsy. II: Methodological considerations on hyper‐ and hypoperfusion

**DOI:** 10.1002/epi4.12833

**Published:** 2023-10-12

**Authors:** Martin Prener, Veronica Drejer, Morten Ziebell, Per Jensen, Camilla Gøbel Madsen, Svitlana Olsen, Gerda Thomsen, Lars H. Pinborg, Olaf B. Paulson

**Affiliations:** ^1^ Neurobiology Research Unit, Department of Neurology Rigshospitalet Blegdamsvej Copenhagen Denmark; ^2^ Department of Neurosurgery Rigshospitalet Copenhagen Denmark; ^3^ Department of Radiology, Centre for Functional and Diagnostic imaging and Research Copenhagen University Hospital Amager and Hvidovre Hvidovre Denmark; ^4^ Epilepsy Clinic, Department of Neurology, Rigshospitalet, Copenhagen, Faculty of Health and Medical Sciences University of Copenhagen Copenhagen Denmark; ^5^ Department of Clinical Medicine University of Copenhagen Copenhagen Denmark

**Keywords:** cerebral blood flow, epilepsy surgery, neuroimaging (in addition to words in title), single‐photon emission computed tomography

## Abstract

**Objective:**

Single‐photon emission computed tomography (SPECT) with the tracer ^99m^Tc‐HMPAO is a method to visualize the cerebral hyperperfusion during an epileptic seizure and thus localize the epileptogenic zone and seizure propagation. Subtraction of interictal from Ictal SPECT Co‐registered to MRI (SISCOM) visualizes areas with relative increases in cerebral blood flow. The purpose of this retrospective study is to explore the added value of visualizing areas of hypoperfusion as well as hyperperfusion, so‐called reversed SISCOM.

**Methods:**

Fifty‐six patients operated for epilepsy who had been investigated with SISCOM were included in the analysis. The patients were divided into two groups based on seizure duration after tracer injection, above or below 30 s. The preoperative SISCOM description was compared to the area of resection and given a concordance score. The 56 SISCOM were recalculated visualizing also areas of hypoperfusion and again compared to the site of resection using the same scale of concordance. The reversed SISCOM were categorized into three subgroups: “Altered Conclusion,” “Confirmed Conclusion,” and “Adds Nothing.” If an area of hyperperfusion had an area of hypoperfusion in close proximity, it was re‐interpreted as noise, thus possibly altering the conclusion. If the areas of hypoperfusion were in the opposite hemisphere it was interpreted as confirming factor. Further the concordance scores from conventional SISCOM and reversed SISCOM was compared to surgical outcome to explore the difference in sensitivity, positive predictive value (PPV), and odds ratio.

**Results:**

In approximately half of the cases reversed SISCOM added additional value, meaning either altered the conclusion or confirmed the conclusion. The sensitivity, PPV, and odds ratio was also better in the subgroup of long, >30 s seizure duration after injection, and got worse in the group with short, <30 s seizure duration after injection.

**Significance:**

Adding reversed SISCOM performed better than conventional SISCOM at predicting good surgical outcome.

**Key points**

- SPECT with the tracer ^99m^Tc‐HMPAO can be used to also visualize hyperperfusion in areas of epileptic activity (SISCOM).
- SPECT with the tracer ^99m^Tc‐HMPAO can also be used to visualize the areas with hypoperfusion, this is called reversed SISCOM.
- Areas of hyperperfusion in close proximity to areas of hypoperfusion was interpreted as noise, and could alter the conclusion of the scan.
- Hypoperfusion in the opposite hemisphere of the hyperperfusion was interpreted as a confirming factor.
- Adding reversed SISCOM performed better than conventional SISCOM at predicting good surgical outcome.

## 1 INTRODUCTION

Several diagnostic tools are used to identify the epileptogenic zone in drug resistant epilepsy patients, but the findings, including MRI, may be negative. In these and other selected cases, ictal measurement of the regional cerebral blood flow (rCBF) distribution by SPECT of ^99m^Tc‐hexamethylpropyleneamine‐oxime (^99m^Tc‐HMPAO) may add to the location of the epileptogenic focus as epileptic activity is accompanied by an increase in blood flow in the involved cortical areas.^1^ ^99m^Tc‐HMPAO is a special radioactive tracer which is retained in brain tissue proportional to the rCBF (see Section 2).^2^, ^3^, ^4^, ^5^

Subtraction Ictal by interictal SPECT and co‐registered to MRI (SISCOM) is a technique to visualize the relative increase in rCBF induced by the epileptic activity. SISCOM requires two separate ^99m^Tc‐HMPAO SPECT scans, one with tracer injection during a seizure (ictal) and one in the resting state (interictal).^6^

SISCOM visualizes traditionally areas of relative increased blood flow at the time the tracer reaches the brain, which might not be the epileptogenic zone but neighboring areas due to seizure propagation. But, is it possible to obtain additional valuable information also visualizing the areas of relative decreased rCBF? If the seizure is short or the injection is too late the tracer might reach the brain in an early post‐ictal phase. The aim of the present study is to explore the added benefit of also visualizing areas of hypoperfusion. For this purpose, we reanalyzed data from our recent study investigating the use of conventional SISCOM.^7^

## 2 METHODS

Below are the key aspects of the method summarized. For further information regarding the method please consider reading part I of the study.^7^

The present evaluation of the result and of methodological evaluation was done retrospectively. The evaluation was approved by Danish Health and Medicines Authority (2019, record number: 3‐3013‐1030/1).

The clinical indication for the scan in each individual patient was determined by the attending epilepsy physician and/or a multidisciplinary clinical team, who were ultimately responsible for making the decisions regarding epilepsy surgery.

### 2.1 Patient inclusion

The presurgical evaluation of the patients is performed at the Epilepsy Hospital in Dianalund or at the Department of Neurology at Rigshospitalet. SISCOM was predominantly used in cases where diagnosis of the epileptogenic focus was difficult and where a seizure duration of at least 30 s could be expected.

The patient material in the present study is the same as in our first publication dealing with predictive values.^7^ In that report we argued for dividing the patients into two groups, group 1 with seizure duration after tracer injection >30 s and group 2 with seizure duration after tracer injection <30 s, because an intravenous (IV) bolus reaches the brain approximately 30 s after injection.^8^ This grouping of the patients was maintained in the present study. Fifty‐eight patients were operated, one of these patients was excluded due to missing follow‐up, and another one was excluded from the present study due to unavailable data. This leaved 56 patients for the present analysis, see Figure S1 and Table S1 both in supplementary material.

The patients had follow‐ups 1 and 2 years after the operation, Figure S1.

### 2.2 Setup and procedures

For the ictal SPECT scan the patient's antiseizure medicine was tapered off. A specialized technologist observed the patient and noted the time of seizure onset, defined by either the beginning of rhythmic ictal EEG discharges or earliest onset of clinical symptoms. At the start of the seizure the technologist immediately injected the radiotracer, ^99m^Tc‐HMPAO intravenously. The end of the seizure was based solely on clinical observation. The technologist timed the entire seizure, see Table S1.

The same procedure was used for the interictal SPECT, except the scan being performed on an outpatient basis. The patients were seizure‐free from clinical seizures for the last 24 h prior to the scan.

### 2.3 SISCOM and reverse SISCOM processing

SISCOM uses the SPECT tracer ^99m^Tc‐HMPAO, a lipophilic tracer that easily crosses the blood–brain barrier (BBB). Once it has crossed the BBB it is rapidly converted to a hydrophilic compound which cannot cross the BBB, and therefore retained in the brain tissue. As the tracer is sufficiently stable the SPECT scan can be postponed a few hours after tracer injection.^9^ A protocol was made for dosage volume in relation to time of injection, planned dosage 900 Mbq. For all groups, ictal and interictal the median tracer dosage was around 917 MBq ^99m^Tc‐HMPAO.

SISCOM analysis was performed using the Analyze software (*Biomedical Imaging Resource, Mayo Foundation*) by subtracting the interictal SPECT from the Ictal SPECT and consequently visualizing areas of hyperperfusion (red/yellow) with a *z*‐score higher than 2, and areas of hypoperfusion (blue) with a *z*‐score lower than −2.^10^, ^11^ For the latter we used the term “reversed SISCOM.”

### 2.4 Preoperative SISCOM scoring and relation to surgical outcome

The preoperative SISCOM description was compared to the area of resection was the same as used in part I of the study.^7^ It was made by MZ and PJ. In order to differentiate a valid focus from background noise, the criterion involved having an approximate size of 2.5 cm^3^ and being situated within the cortex. A scale from 2 to −1 was used: 2 = SISCOM hyperperfusion in the same hemisphere and lobe. 1 = SISCOM hyperperfusion in same hemisphere and neighboring lobe or there are two foci, one being in the correct hemisphere and lobe and one focus in the opposite hemisphere. 0 = SISCOM showed no‐foci. −1 = SISCOM hyperperfusion in opposite hemisphere or hyperperfusion in same hemisphere but not neighboring lobes for example, occipital lobe with surgical focus in the frontal lobe. The concordance scores where then compared to the surgical outcome using the Engel score I to IV after respectively 1‐ and 2‐years follow‐up. In the present study we used the same form of analysis.

### 2.5 Reanalysis of SISCOM including areas of hypoperfusion and relation to surgical outcome

The reanalysis used the same concordance scale as described above for the preoperative evaluation, but modified according to the presence of relatively hypoperfused areas. The reanalysis of the reversed SISCOM was made by OP and MP. The 56 SISCOM could be categorized into three subgroups: “Altered Conclusion,” “Confirmed Conclusion,” and “Adds Nothing.” Altered conclusion was used when the expected focus had areas of hypoperfusion in close proximity or when there was more than one hyperperfusion (red/yellow) focus, but one or more had areas of hypoperfusion (blue) in close proximity. Confirmed was defined as hypoperfusion (blue) exclusively or dominantly in the opposite hemisphere of the hyperperfusion (red/yellow) focus or in no‐foci cases hypoperfusion (blue) in close proximity to areas of hyperperfusion (red/yellow). The subgroup “Adds Nothing” is self‐explanatory.

### 2.6 Comparison of “conventional SISCOM” and “reversed SISCOM”

The new concordance scores and the surgical outcome were used to calculate, sensitivity, positive predictive value (PPV), and odds ratio at the 1 and 2‐year follow‐up using a two‐by‐two matrix. The two axes in the matrix were good and bad surgical outcome and good and bad concordance between areas of resection and the SISCOM. Good surgical outcome was defined as Engel I, freedom from disabling seizure. Good concordance was defined as 1 or 2, bad concordance was defined as 0 or −1, the same as in part I of the study. The sensitivity, PPV, and odds ratio were compared to the ones shown in “Ictal and interictal SPECT with ^99m^Tc‐HMPAO in presurgical epilepsy. I: Predictive value and methodological considerations”,^7^ which are also showed in Table 2. Lastly, we also calculated the ratio of odds ratios between group 1 and 2 at the 2‐year follow‐up, like we did in part I. We used the Zelen test to calculate a *P*‐value for the ratio of odds ratios between group 1 and 2.

## 3 RESULTS

The mean age of the patient in group 1 (>30 s seizure duration after IV tracer injection) and group 2 (<30 s seizure duration after IV tracer injection) were 29 years (6–57) and 19.5 years (5–49) respectively. The median seizure frequency before SPECT was 3.5 (1.5–150) and 4 (2–240) seizures per month, respectively for the two groups. Previous brain surgery for epilepsy had been performed in 8 (21%) in group 1, none in group 2. Furter demographic data are given in Table S1 in supplementary material.

For *group 1* adding the regions with relative flow decrease to the SISCOM evaluation changed the conclusion in 4 of 39 cases, which is slightly above 10%. The conclusion was further confirmed in 13 of the cases, 33%. In the remaining 22 cases reversed SISCOM added no further information. For group 1 reversed SISCOM added additional valuable information in slightly below half of the cases, see Table 1.

**TABLE 1:** Shows the number and percentage of the three subcategories of the reanalysis.

Group 1, >30 s seizure duration after injection		
	No. (39)	Percentage
Altered conclusion	4	10.3
Confirms conclusion	13	33.3
Adds nothing	22	56.4

Group 2, <30 s seizure duration after injection		
	No. (17)	Percentage
Altered conclusion	5	29.4
Confirms conclusion	5	29.4
Adds nothing	7	41.2

For *group 2* the conclusion was altered in 5 of 17 cases. Another five of the cases the conclusion was further confirmed and the remaining seven cases reversed SISCOM added nothing. For group two reversed SISCOM added additional valuable information in approximately 60% of the cases, see Table 1.

In Table 2 the sensitivity, PPV and odds ratio is shown for both the conventional SISCOM and the adding reversed SISCOM analysis in the preoperative evaluation. For *group 1* both at the 1‐year and the 2‐year follow‐up the sensitivity was unchanged. The PPV was increased with circa three percentage points at both follow‐ups. The odds ratios were also increased. At the 1‐year follow‐up from 2.38 to 3.17 and at the 2‐year follow‐up from 5.71 to 7.5.

**TABLE 2:** Show a side‐by‐side comparison of the sensitivity, PPV, and odds ratio with *p*‐values for conventional SISCOM as well as for Reversed SISCOM.

	>30 s seizure duration after injection	
	Conventional SISCOM	Reversed SISCOM
1 year		
Sensitivity	76.0	76.0
PPV	70.4	73.1
Odds ratio	2.38 (CI_95%_: 0.60–9.64)	3.17 (CI_95%_: 0.79–12.75)
	*(p = 0.287)*	*(p = 0.1574)*
2 year		
Sensitivity	83.3	83.3
PPV	74.1	76.9
Odds ratio	5.71 (CI_95%_: 1.30–25.03)	7.5 (CI_95%_: 1.69–33.27)
	*(p = 0.031)* *	*(p = 0.0126)* *

	<30 s seizure duration after injection	
	Conventional SISCOM	Reversed SISCOM
1 year		
Sensitivity	40.0	22.2
PPV	40.0	25.0
Odds ratio	0.22 (CI_95%_: 0.03–1.71)	0.095 (CI_95%_: 0.01–0.90)
	*(p = 0.188)*	*(p = 0.0567)*
2 year		
Sensitivity	50.0	33.3
PPV	55.6	42.9
Odds ratio	0.75 (CI_95%_: 0.11–5.24)	0.375 (CI_95%_: 0.05–2.88)
	*(p = 1)*	*(p= 0.6145)*

* Meaning significant with an alpha level of 0.05.

For *group 2* the sensitivity, PPV and odds ratio at both the 1‐ and 2‐years follow‐ups was lower when using the concordance scores from added reversed SISCOM compared to conventional SISCOM. Comparing conventional SISCOM to reversed SISCOM at the 1‐year follow‐up the sensitivity went from 40 to 22.2, the PPV from 40 to 25, and the odds ratio from 0.22 to 0.095. At the 2‐year the sensitivity went from 50 to 33.3, the PPV from 55.6 to 42.9 and the odds ratio from 0.75 to 0.375, see Table 2.

### 3.1 In the following a few illustrative examples will be shown

**FIGURE 1:**
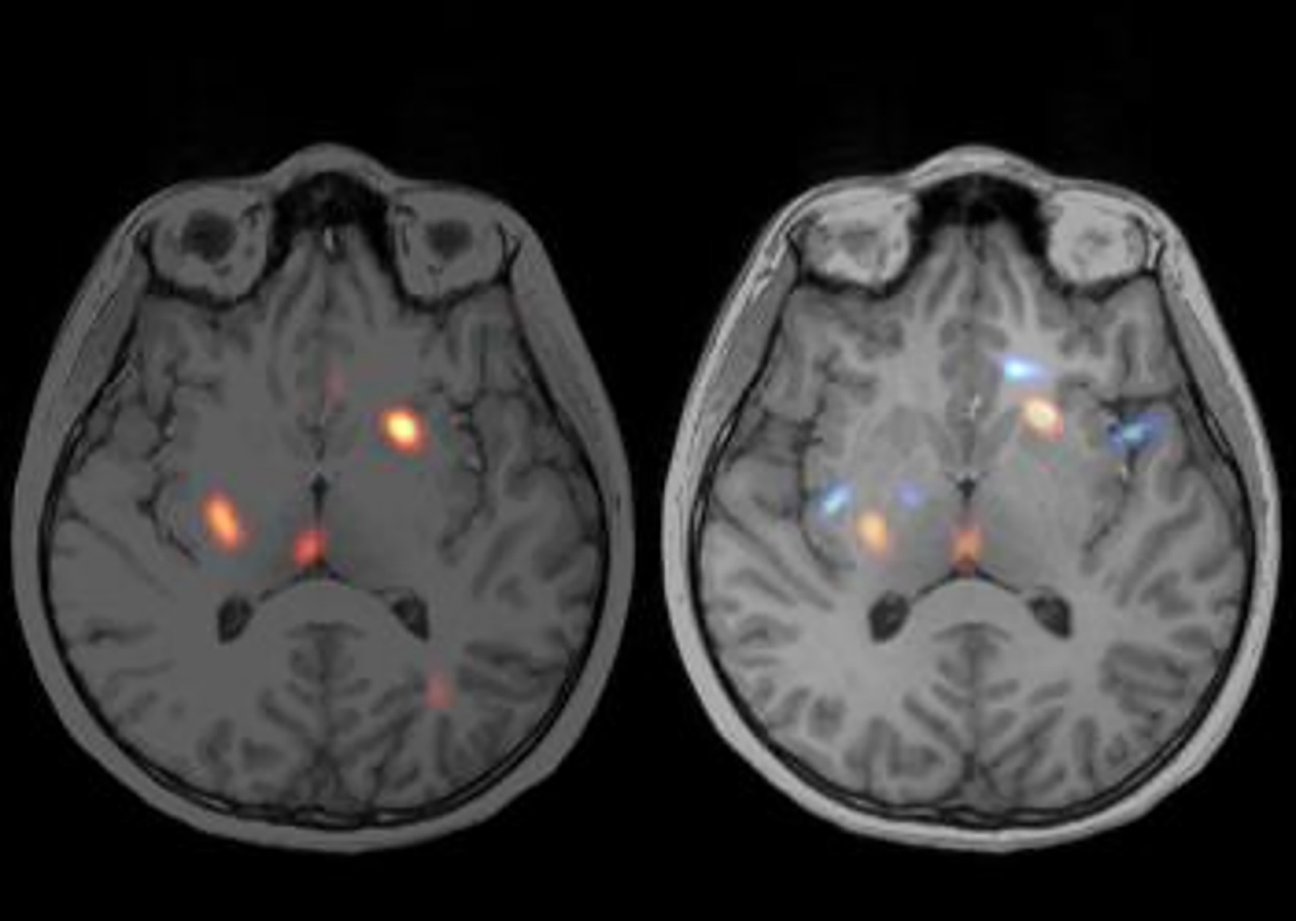
Visualizing hypoperfused areas changed the conclusion from focus to no focus. (A) Left: SISCOM with only hyperperfused (red/yellow) areas. (B) Right: with both hyper‐(red/yellow) and hypoperfusion (blue) areas. The patient had a seizure duration after tracer injection of 11 s and an injection latency of 17 s. The outcome of the surgery was Engel I, resection in left frontal lobe.

In Figure 1 the conclusion was altered from a focus in anterior part of insula to no‐foci. Figure 1A (left) shows the preoperative conventional SISCOM, with only areas of hyperperfusion (red/yellow). The conclusion of the conventional SISCOM was a focus in the anterior part of insula. Figure 1B (right) with areas of hypoperfusion (blue) added shows that the focus in the anterior part of the left insula is surrounded by two blue areas, thus altering the conclusion to no‐foci. The patient was operated in the left frontal lobe and had Engel I as surgical outcome. The seizure duration after injection was 11 s, meaning it did not qualify as an optimal scan. Despite the good surgical outcome of this case, it shows how easily a scan can be misinterpreted.

**FIGURE 2:**
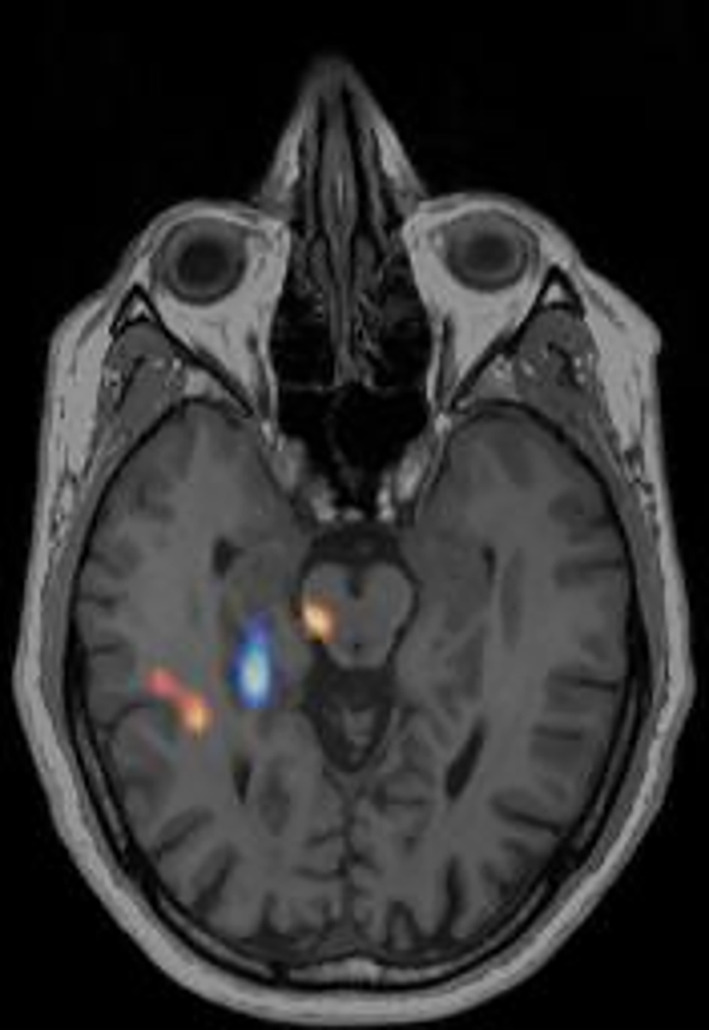
Visualizing hypoperfused areas changed the conclusion from right temporal focus to no focus. SISCOM with both hyper‐(red/yellow) and hypoperfusion (blue) areas. The patient had a seizure duration after tracer injection of 0 s and an injection latency of 195 s. The outcome of the surgery was Engel I, resection in left frontal lobe.

In Figure 2 the conclusion was altered from right temporal lobe to no‐foci. There were several other foci spread out primarily subcortical. There is an area of hypoperfusion (blue) in close proximity and thus the area of hyperperfusion (red/yellow) is interpreted as noise. The area of resection was right frontal lobe and the surgical outcome was Engel I. The seizure duration after injection was 0 s for this patient, meaning the scan would be classified as a poor‐quality scan due to the low seizure duration after injection. The focus was in the correct hemisphere and in the neighboring lobe, but it is interpreted as a coincidence due to the area of hypoperfusion (blue) right next to it.

**FIGURE 3:**
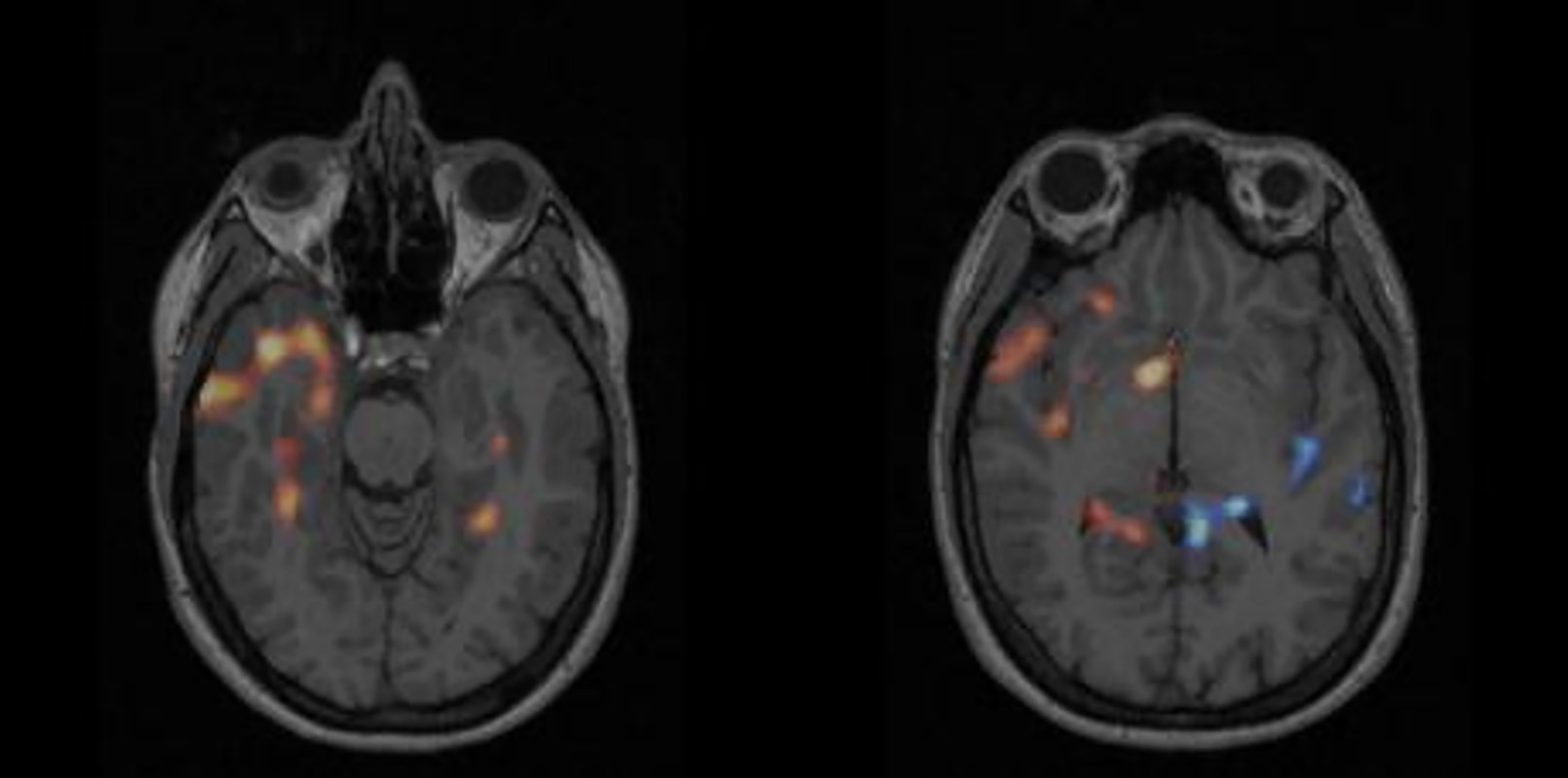
Visualizing hypoperfused areas confirmed the conclusion of a right temporal lobe focus. Left: SISCOM with only hyperperfused (red/yellow) areas. Right: with both hyper‐(red/yellow) and hypoperfusion (blue) areas. The patient in had a seizure duration after tracer injection of 75 s and an injection latency of 22 s. The outcome of the surgery was Engel I, resection in right temporal lobe.

In Figure 3 and Figure S2 the preoperative conclusion was confirmed. In both cases there was a clear temporal focus, a seizure duration after injection of more than 30 s, and a low injection latency. These are optimal scan preconditions. In Figure 3 the hypoperfused areas are located more cranially than the hyperperfused areas in focus. Figure S2 visualize both hyper‐ and hypoperfused areas. In both figures there are areas of hypoperfusion/blue areas in the contralateral hemisphere. Both patients were operated in the hyperperfused areas and had good surgical outcome, Engel I at both the 1 and 2‐year follow‐up. In these cases, the additional hypoperfusion further confirmed the conclusion of the conventional SISCOM.

**FIGURE 4:**
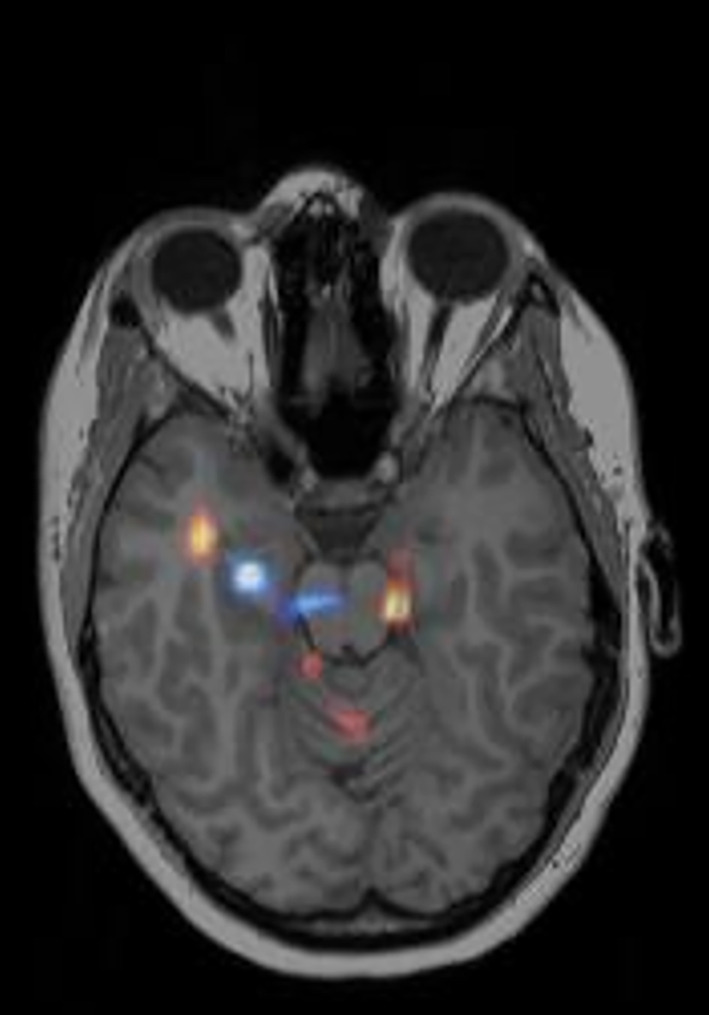
Visualizing hypoperfused areas confirmed the conclusion of no focus. SISCOM with both hyper‐(red/yellow) and hypoperfusion (blue) areas in the same lobe. The patient had a seizure duration after tracer injection of 17 s and an injection latency of 26 s. The outcome of right frontal lobe resection was Engel II.

In Figure 4 the preoperative conclusion, no‐foci, was further confirmed. As shown, there is an area of hyperperfusion in the right temporal lobe. This might have been interpreted as an epileptogenic focus due to the relative high occurrence of temporal epilepsy. It should be noted that the area is relatively small and there are several other areas of that size throughout the scan, thus the preoperative conclusion of the scan was no‐foci. The area of hypoperfusion (blue) is in close proximity to the area of hyperperfusion (red/yellow) thus confirming the conclusion of no‐foci. The seizure duration after injection was 17 s, which means the tracer bolus did not reach the brain during the active seizure.

## 4 DISCUSSION

An epileptic seizure induces hyperperfusion in the affected area of the brain. This hyperperfusion originates from the epileptogenic zone and propagates with the epileptic activity to neighboring areas. Hyperperfused (red/yellow) areas visualized on SISCOM that are in close proximity to an area of hypoperfusion (blue) would most likely be noise and not as a true sign of epileptic activity. Thus, SISCOM visualizes brain areas with the 2.5% (two *z*‐scores) highest increases in ictal blood flow compared to the baseline interictal scan. In case of no focal changes, only physiological fluctuation, the hyperperfused and hypoperfused areas might be randomly spread out.

Still, we have to take into consideration that a hypoperfused area might also be related to an early postictal phase, as might be present if the seizure is either very short or the injection is late. Thus, 2 min post seizure the blood flow largely normalizes,^1^ but the cerebral blood flow between the end of the seizure and the 2 min post seizure is still a mystery. This gives rise to some reflections regarding what the hypoperfused areas reflects, especially in group 2, where the tracer reached the brain post seizure. Could it in some instances reflect post ictal neuronal depression in the epileptogenic zone and thus be part of the area just involved in the epileptic seizure, and thus support the presence of a seizure zone. A study by Gaxiola‐Valdez et al^12^ scanned 21 epilepsy patient with an MRI angiography within 90 min of a seizure. This scan was compared to a baseline scan and they found 15 of the 21 had postictal hypoperfusion. This correlates well with our general hypothesis that the bolus of the injected tracer should ideally arrive at the brain while there is still an active seizure going on.

Cho et al^13^ showed that a temporal seizure might swiftly propagate to the contralateral side and cause hyperperfusion there and hypoperfusion in the ipsilateral side using SISCOM, and there is al a difference in the seizure propagation from left and right temporal lobes.^14^ We experienced this with one of the patients with good surgical outcome. This patient in our study had a cystic intraventricular tumor in the left anterior horn and a SISCOM focus in the right temporal lobe. The SISCOM scan of this patient was regarded as high quality following the criteria previously described. The patients in the study by Cho et al^13^ all had unilateral hippocampal sclerosis. Overall, this phenomenon did not affect our study.

Lee and co‐workers also points out that an area with mixed neighboring hyper‐ and hypo‐ perfused foci could represent a phase where part of the tissue where postictal simultaneous to other being ictal.^15^ If both hyper‐ and hypoperfused areas were in close proximity it might represent “surround inhibition.” We have not observed cases where we would consider a hypoperfused area to represent an area with postictal depression.

### 4.1 Comments on data

Overall reversed SISCOM added additional valuable information in 27 of the total 56 cases. The percentage was slightly higher in group 2 (seizure duration after tracer injection <30 s) compared to group 1 (seizure duration after tracer injection >30 s). In group 2 several conclusions were no‐foci, probably due to the short seizure duration after injection, which means that the tracer reached the brain after the end of the seizure. This is illustrated in Figure 4. In group 1, only 10% of the conclusions were altered. This most likely reflects the longer seizure duration after injection. In this group the reversed SISCOM was in many cases further useful in confirming the conclusion, for example, when hypoperfusion was only present in the opposite hemisphere.

The sensitivity in group 1 was unchanged but the PPV and the odds ratio was better when including reversed SISCOM. In group 2 it was the opposite, meaning the sensitivity, PPV and odds ratio was lower with reversed SISCOM. Thus, in group 1 with optimal scans, overall including reversed SISCOM was better than conventional SISCOM at predicting good surgical outcome. In group 2 with suboptimal scans reversed SISCOM was even poorer than conventional SISCOM. The ratio of odd ratios (7.5 and 0.375, respectively) comparing group 1 and group 2 at the 2‐year follow‐up was 0.05 with (*P = 0.051*) using the Zelen test. Though not significant, it shows a lower ratio of odds ratio then that of conventional SISCOM at the 2‐year follow‐up. This difference between group 1 and 2 further emphasis the main point of part I of the study^7^: to use a 30 s cut‐off in seizure duration after injection as a valuable tool to secure good quality SISCOM.

## 5 CONCLUSION

Despite only 56 cases studied with reversed SISCOM, we consider this methodological add‐on to provide valuable information, not only in the cases where the conclusion of SISCOM scan was altered. It made the investigators more confident in their conclusion in one third of the cases, as artifacts and inconsistent changes become more evident. This is further supported by our analysis of SISCOM in several non‐operated patients not included in the present study.

In cases where the conclusion was further confirmed the areas of hypoperfusion (blue) were typically at a distance from the hyperperfused (red/yellow) areas, that is, the contralateral hemisphere, thus confirming that the hyperperfusion represents a true signal/epileptic activity.

To draw a definitive conclusion based on the reanalysis part of our study would be premature. The relevance of including the areas with relative flow decrease in the prospective evaluation calls for future research with optimal SPECT scans meaning, duration of seizure after injection >30 s, injection latency no more than 45 s after seizure start and adding reverse SISCOM so the parts of the brain with the least radiotracer deposition becomes visible as well.

## AUTHOR CONTRIBUTIONS

Martin Prener: Analyzed data, drafted manuscript. Veronica Drejer: analyzed data. Morten Ziebell: Described the SPECT scans prior to surgery. Per Jensen: Described the SPECT scans prior to surgery. Camilla Gøbel Madsen: Described MR scans. Svitlana Olsen: Performed the ictal and interictal scans and generated the SISCOM images. Gerda Thomsen: Performed the ictal and interictal scans and generated the SISCOM images. Lars H. Pinborg: Recruited the patients. Olaf B. Paulson: Planed and supervised the analysis of the material. Drafted manuscript. All authors participated in drafting the final version of the manuscript.

## CONFLICT OF INTEREST STATEMENT

None of the authors has any conflict of interest to disclose. We confirm that we have read the Journal's position on issues involved in ethical publication and affirm that this report is consistent with those guidelines.

## CLINICAL TRIAL STATEMENT

The present study is a retrospective evaluation of the use of SPECT with the tracer ^99m^Tc‐HMPAO in presurgical investigation of patients with epilepsy as introduced in the clinical work in the years 2010–2019. It was introduced for clinical purposes and not registered as a clinical trial.

## Supporting information


Appendix S1.

## Data Availability

Data can be obtained by contacting the authors. The access to and use of the data must be in accordance with the rules of the Danish legislation and must be approved according to the Danish Data Protection Agency's rules.
