# Supplementary material for: Ictal and interictal SPECT with 99mTc‐HMPAO in presurgical epilepsy. II: Methodological considerations on hyper‐ and hypoperfusion
Source: Epilepsia Open. 2023 Oct 12;8(4):1503–11. doi: 10.1002/epi4.12833 (PMC10690685; doi:10.1002/epi4.12833)
Supplement: Supplementary file 1 — Appendix S1. [file EPI4-8-1503-s001.zip › Table S1.docx]

**Supplementary material**

**Figure S1** shows a flow-chart of patient inclusion.

**Figure S2**: *Visualizing hypoperfused areas confirmed the conclusion of a left temporal lobe focus.* SISCOM with both hyper-(red/yellow) and hypoperfusion (blue) areas. The patient had a seizure duration after tracer injection of 55s and an injection latency of 24s. The outcome of the surgery was Engel I, resection in left temporal lobe.

| **Table S1** Patient, Clinical and basic SPECT data | Group 1 (>30s) | Group 2  (<30s) |
| --- | --- | --- |
| No. patients | 39 | 17 |
| Age at SPECT in years | 29 (6-57) | 19 (5-49) |
| Sex female (%) | 21 (54) | 9 (50) |
| Median seizure frequency before SPECT, no. of seizures/month | 3.5 (1.5-150) | 4 (2-240) |
| No. of patients with history of previous brain surgery (%) | 8 (21) | 0 (0) |
| No. of patients with one or more MRI lesions suspected to be epileptogenic, (%) | 23 (59) | 7 (42) |
| Duration of seizure after tracer injection in seconds* | 76 (30-276) | 13 (0-28) |
| Injection latency, time from seizure onset to injection in seconds* | 20 (9-40) | 17 (8-195**) |
| Injected tracer dose Ictal, MBq | 925 (211-1075) | 888 (160-999) |
| Injected tracer dose Interictal, MBq | 921 (244-1088) | 899 (178-1043) |

Table S1 shows basic patient data, clinical and SPECT data for the 39 patients included. Continuous variables are shown as median and range in parenthesis. *Seizure onset: based on EEG or clinical symptoms, what comes first; Seizure end: based on clinical symptoms. ** The second latest injections latency was 46s.
