# Supplementary figures and images for: Ictal and interictal SPECT with 99mTc‐HMPAO in presurgical epilepsy. II: Methodological considerations on hyper‐ and hypoperfusion
Source: Epilepsia Open. 2023 Oct 12;8(4):1503–11. doi: 10.1002/epi4.12833 (PMC10690685; doi:10.1002/epi4.12833)

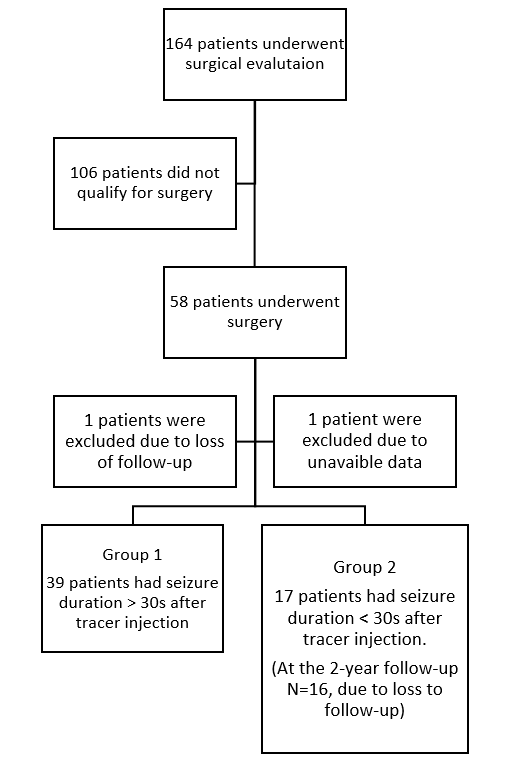

Supplement: Supplementary file 1 — Appendix S1. [file EPI4-8-1503-s001.zip › SISCOM_II_Figure_SI.tif]

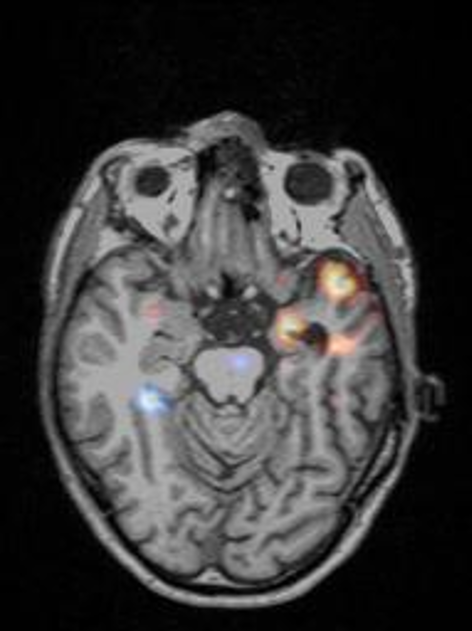

Supplement: Supplementary file 1 — Appendix S1. [file EPI4-8-1503-s001.zip › SISCOM_II_Figure_SII.tif]
